# Green Synthesis of Phosphorous-Containing Hydroxyapatite Nanoparticles (nHAP) as a Novel Nano-Fertilizer: Preliminary Assessment on Pomegranate (*Punica granatum* L.)

**DOI:** 10.3390/nano12091527

**Published:** 2022-05-01

**Authors:** Hala M. Abdelmigid, Maissa M. Morsi, Nahed Ahmed Hussien, Amal Ahmed Alyamani, Nawal Abdallah Alhuthal, Salim Albukhaty

**Affiliations:** 1 Department of Biotechnology, College of Science, Taif University, P.O. Box 11099, Taif 21944, Saudi Arabia; a.yamani@tu.edu.sa; 2 Department of Biology, College of Science, Taif University, P.O. Box 11099, Taif 21944, Saudi Arabia; m.moasa@tu.edu.sa; 3 Department of Chemistry, College of Science, Taif University, P.O. Box 11099, Taif 21944, Saudi Arabia; nawal.h@tu.edu.sa; 4 Department of Chemistry, College of Science, University of Misan, Maysan 62001, Iraq

**Keywords:** hydroxyapatite nanoparticles, nano-fertilizer, *Punica granatum*, green synthesis

## Abstract

Nano-fertilizers are innovative materials created by nanotechnology methodologies that may potentially replace traditional fertilizers due to their rapid absorption and controlled distribution of nutrients in plants. In the current study, phosphorous-containing hydroxyapatite nanoparticles (nHAP) were synthesized as a novel phosphorus nano-fertilizer using an environmentally friendly green synthesis approach using pomegranate peel (PPE) and coffee ground (CE) extracts. nHAPs were physicochemically characterized and biologically evaluated utilizing the analysis of biochemical parameters such as photosynthetic activity, carbohydrate levels, metabolites, and biocompatibility changes in *Punica granatum* L. Cytocompatibility with mammalian cells was also investigated based on MTT assay on a Vero cell line. Dynamic light scattering (DLS) and zeta potential analysis were used to characterize the nHAPs for size and surface charge as well as morphology using scanning electron microscopy (SEM) and transmission electron microscopy (TEM). The nHAPs were found to have different shapes with average sizes of 229.6 nm, 120.6 nm (nHAPs_PPE) and 167.5 nm, 153 nm (nHAPs_CE) using DLS and TEM, respectively. Overall, the present results showed that the synthesized nHAPs had a negative impact on the selected biochemical, cytotoxic, and genotoxic parameters, indicating that the evaluation of nHAP synthesized by this approach has a wide range of applications, especially as a nano-fertilizer.

## 1. Introduction

Nanotechnology in agriculture involves the controlled delivery of certain substances, primarily pesticides and fertilizers, but uptake and use efficiency, as well as effects of nanoparticles on metabolic functions in plants, vary among species. Plants are examined for their response to metal nano-fertilizers such as Cu, Mn, Zn, and Fe, as well as chemicals such as Al, Ce, La, and Ti as nanoparticles [1]. Subsequently, worldwide metal nanoparticle production has grown exponentially over the past decade, with 58,000 metric tons projected by 2020 [2]; meanwhile, macronutrient research is restricted, although these elements lead to global crop production [3]. Phosphorus deficiency is a common factor hindering yield and crop quality worldwide [4], and sustaining adequate crop production will primarily require higher demand for phosphate fertilizers as P is an essential element for plant growth and development.

Elemental phosphorus (P) is taken up by plants in the form of plant-accessible water-soluble Pi salts, which are applied to fields as mono and diammonium Pi (MAP, DAP) and triple super Pi (TSP). However, only about 20% of the phosphate applied to fields is used by plants during a growing season [5], as some of it forms complexes with soil Al, Ca, and Fe oxides and precipitates in plant-inaccessible forms such as phosphates of Fe, Ca, and Al. Furthermore, large amounts of soluble Pi are being lost through agricultural waste into local water bodies, where they contribute to eutrophication and cause algae blooms, which are severely harmful to aquatic ecosystems [6]. In addition, phosphate fertilizers are made from mineral rock Pi, which is a finite non-renewable resource whose reserves are declining [7]. Alternative methods of fertilizing are worth exploring in the short term, even though in the long run, plants can acquire Pi more readily. Therefore, increasing P uptake and utilization is essential for agricultural crops such as pomegranate, while providing nano-fertilizers with phosphorus may be an alternative. Spraying nano-fertilizer requires less energy and costs less than chemical fertilizers because they have high surface areas, high adsorption capacity, and controlled release kinetics.

Improved phosphorous use efficiency can be achieved through better land use and erosion prevention, soil maintenance, improved fertilizer recommendations, fertilizer placement methods, and the promotion of mycorrhizas [8]. A potential strategy to increase the efficiency of using P fertilizers may be using synthetic nano-hydroxyapatite [Ca_10_(PO_4_)_6_(OH)_2_; nHAP]. Since nHAP is not water-soluble, this could limit the reactions in soil, reduce soil precipitation and/or adsorption on soil colloids, and increase plants’ access to it [9]. nHAP is capable of increasing P use efficiency through mechanisms such as targeted delivery, slow or controlled release, and low molecule size. Hydroxyapatite (HA) nanoparticles are ranked as one of the most popular candidates in agronomic applications which can provide phosphorous nutrients. Many of the present studies on nHAP are focused on its biomedical applications due to its excellent biocompatibility and bioactivity, while potential agriculture applications have not been adequately resolved [10,11]. Kottegoda et al. [10] produced slow-release fertilizer from urea-modified hydroxyapatites as an alternative fertilizer and concluded that it can potentially increase soybean yields when grown in peat–perlite mixtures.

In the literature [12], several methods for creating nHAP have been described. These include wet chemical deposition, biomimetic routes, sol–gel methods, and hydrothermal methods. These conventional methods require the overt use of many chemicals to achieve a controlled synthesis, but synthesized nanomaterials usually have high toxicity [12]. As a result, current nanotechnology focuses on environmentally friendly green synthesis processes [13], where biological systems such as bacteria, fungi, and plant extracts show the potential to serve as nano-manufacturing units [13,14]. The ability of biological systems to control inorganic crystal structures, phases, orientations, and nanostructural topography is known as bio-mineralization. Bio-mineralization is considered one of the biomimetic routes for the preparation of nanostructured powders and coatings for HAP materials that has numerous advantages over other solid-state and wet chemical methods [15,16]. There are few reports in the literature on bio-mineralization used for the synthesis of nHAP, despite its many advantages over other available methods [17,18,19,20].

In this regard, foliar fertilizer application is generally preferred because the amount of fertilizer applied per hectare is very small, and it reduces the number of passes of application, thus preventing soil compaction. It proved most effective and economical to improve plant growth [11,21] and is also less likely to pollute groundwater.

Pomegranates (*Punica granatum* L.; Punicaceae) are fruit-bearing deciduous shrubs and small trees native to Iran and well-adapted to Mediterranean climates that are grown widely around the world [22]. These qualities make pomegranates an attractive candidate fruit crop for cultivation on marginal lands and thus resource-poor farming. Pomegranates are mainly consumed as fresh fruit, but they are also used in jams, juices, vinegar, and jellies [23]. Pomegranates’ consumption has increased recently due to their organoleptic qualities and health benefits [24]. Researchers have employed nanoparticles to improve the efficacy and biocompatibility of a wide range of products, which could be used in a variety of biomedical applications, including anticancer chemotherapeutics, drug delivery, wound dressing, nano biosensors, and agriculture [25,26,27,28,29].

Saudi Arabia is a major producer of pomegranate (*Punica granatum*), as this fruit contains polyphenols, which have shown excellent antioxidant properties, making this fruit popular in the food industry and in traditional medicine for the treatment of various diseases.

As a preliminary assessment, the purpose of this study was to evaluate the effects of foliar application of green factorized hydroxyapatite nanoparticles as phosphorous nano-fertilizers on biochemical parameters (photosynthetic pigments, total soluble carbohydrate), and biocompatibility with biological systems. *Punica granatum* L. was used as model plant species. To reduce the effects of interactions between nanophosphorus fertilizer and other nutrients, a solution containing no other nutrients was used. P being chosen as its deficiency adversely affects crop production globally. The specific objectives are to (1) study the characteristics of nHAP created via two different biogenic routes based on plant extracts of pomegranate fruit peel and solid coffee ground; (2) study the potential of nHAP as a nano-fertilizer on some biochemical parameters including photosynthetic activity, carbohydrate levels, metabolites of *Punica granatum* plants; (3) evaluate the biocompatibility interactions of nHAP with biological systems including mammalian and plant cells as well. 

## 2. Materials and Methods

### 2.1. Cells and Chemicals

The tricalcium phosphate (Ca_3_P_2_O_8_) was purchased from Sigma-Aldrich, Saint-Louis, MO, USA. Vero E6 (ATCC^®^ CRL-1586™) was obtained from the American Type Culture Collection (ATCC, Manassas, VA, USA).

### 2.2. Plant Extracts Preparation and Green Synthesis of Phosphorous Nanoparticles

The aqueous extracts of *Punica granatum* L. peel (PPE), along with the coffee ground extract (CE), were prepared according to our previous study [30]. The green synthesis of phosphorous nanoparticles was conducted according to Tarafdar et al. [31], with a few modifications. The tricalcium phosphate solution (90 mL, 1 mM, Ca_3_P_2_O_8_) was mixed for 72 h with 10 mL of each sample extract. A low-speed shaker at 150 rpm was used to stir the mixture for 72 h until a color change was observed, as illustrated in Figure S1 (Supplementary File). The mixtures were centrifuged for 30 min at 6000 rpm, the pellets were washed with dH_2_O, and then, phosphorous nanoparticles were collected by centrifugation. Purified pellets (nHAPs_PPE) and (nHAPs_CE) were then placed in the oven (60 °C) for a set amount of time, after which they were scraped for further analysis.

### 2.3. Characterization of Phosphorous Nanoparticles

#### 2.3.1. UV–VIS Spectroscopy

With a UV–VIS-NIR spectrophotometer (UV-1601, Shimadzu, Japan), the absorption spectra of biologically synthesized phosphorous nanoparticles (nHAPs_PPE, and nHAPs_CE) were measured in the range of 200–800 nm.

#### 2.3.2. Surface Morphology Using SEM, TEM, and XRD

The surface morphology of nHAPs_PPE and nHAPs_CE was determined by scanning electron microscope (SEM), transmission electron microscopy (TEM), followed by X-ray diffractometer (XRD). Before SEM examination, dry nHAPs powder was coated with gold for 10 min using a Cressington Sputter Coater (108auto, thickness controller MTM-10, Essex, UK). Coated nanoparticles were scanned by SEM (JEOL JSM-639OLA, Analytical Scanning Electron Microscope, EMU, Taif University) at 20 kV at ×3000 (scale bars = 5 μm) magnification. Nanoparticle shape was also determined by TEM. The samples were prepared by placing a drop of a solution of NPs directly on a copper grid, and imaging was performed using a JEOL-JSM-1400 PLUS (JEOL Tokyo, Japan) at 100 kV. XRD was used to confirm nanoparticle structure (30 kV, and 100 mA, and spectra were recorded using CuKα radiation at a wavelength of 1.5406 Å in the 2θ = 20–80°). Patterns of XRD were plotted by OriginLab software^®^ (2018) and compared with JCPDS Card No. 89-6439.

#### 2.3.3. Dynamic Light Scattering (DLS) Analysis

This technique was set up to determine nanoparticles’ size and their surface charge. A freshly prepared suspension of PNPs_PPE, and PNPs_CE in 0.9% saline solution was sonicated (30 min) at high speed to prevent their aggregations before evaluation. A particle size analyzer (Zetasizer Nano ZS, Malvern Instruments Ltd., Malvern, UK) was used at detection angle = 90°.

#### 2.3.4. Fourier Transform Infrared Spectroscopy (FTIR)

To identify the possible functional groups responsible for nHAPs synthesis, capping, and stabilization, Fourier Transform Infrared Spectroscopy (FTIR, Agilent technologies, Santa Clara, CA, USA) was carried out at 450 to 4000 cm^−1^.

### 2.4. Cytotoxicity MTT Assay and IC_50_ Determination

Culture of monkey kidney cells, Vero E6 in DMEM-high glucose (Sigma), supplied with 10% FBS at a concentration of 1 × 10^5^ cells/mL was conducted in triplicate in serum-free media for 24 h and then treated with nHAPs_PPE and nHAPs_CE at serial concentrations of (50, 100, 150, 200, 250, 300, 350, 400, and 450 μg/mL), separately. The culture media were discarded after 24, 48, and 72 hrs of incubation and 50 μL of 3-(4,5-Dimethylthiazol-2-yl)-2,5-diphenyltetrazolium bromide (MTT, 0.5 mg/mL PBS) was added to each well, which was followed by incubation for 4 h (37 °C, 95% humidified air, and 5% CO_2_). After this, each of the wells was given a final addition of 50 μL of DMSO. An ELISA microplate reader at a wavelength of 570 nm was used to measure the results after shaking the plates for 10 min [32]. Untreated cells were used as control while DMEM-high glucose medium was used as a blank. The absorbance values were converted into percentages of cell viability by using the following formula:

Cell viability (%) = Mean OD of treated cells/Mean OD of control (untreated cells) × 100

The data were plotted using the Microsoft Excel program, and the IC50 values (50% inhibition concentration) at different nHAPs were determined using AAT Bioquest^®^ 2019 as IC50 Calculator.

### 2.5. Plant Materials and nHAPs Applications

#### 2.5.1. Experiment Design

Fruits of *Punica granatum* were obtained from a local nursery in Taif farms (Taify cultivar). Sodium hypochlorite solution (5%) has been used for surface sterilization of the pomegranate seeds for 1 min after the seeds were rinsed with distilled water two times; then, 5 seeds were grown in each pot (30 cm in diameter} filled with clay and sand with the ratio of 2:1 in each pot with the same spaces between the seeds at 25–28 °C in the greenhouse. Tap water was used for the irrigation of the seeds.

The experimental study was carried out based on a randomized complete block design including 5 treatments plus control in three replicates, with each block consisting of 60 plants (5 plants per treatment). Experiments sets were conducted simultaneously using the four concentrations (50, 100, 500, 1000 mg/L) of nHAPs, chemical fertilizer (NPK), and control.

#### 2.5.2. Treatments

During the 30th and 37th days after sowing, 30 mL of phosphorus sources as NPK (chemical fertilizer, 4 g/L) and the above-mentioned hydroxyapatite nanoparticles (nHAP) concentrations were sprayed onto the leaves of plants (25 cm tall) per pot weekly. Hand-held sprayers were used for applying each foliar treatment separately until the leaves were fully humid; the sprays were stopped just before dripping. During the foliar treatments, pot surfaces were covered with a plastic film to prevent any soil contact with the NPs. The nHAPs were formulated with colloidal liquid so that they are more easily absorbed by cells. Pots are maintained in field conditions and watered regularly. Plants were harvested 45–50 days after sowing for analysis. Unfertilized plants were used as control.

#### 2.5.3. Determination of Photosynthetic Pigments

Photosynthetic pigments (chlorophyll a, chlorophyll b, and carotenoids) were determined in leaf extracts using spectrophotometric analysis [33]; leaves were extracted in 85% (*v*/*v*) acetone and centrifuged at 4000× *g* for ten minutes. To measure spectrophotometric absorbance, the supernatant was diluted to an appropriate concentration with 85% aqueous acetone, and the absorbance was measured against a blank of pure 85% aqueous acetone measured at three wavelengths of 452, 644, and 663 nm with the following equations:

Chlorophyll a (μg/mL) = 10.3 E663 − 0.918 E644

Chlorophyll b (μg/mL) = 19.7 E644 − 3.87 E663

Carotenoids (μg/mL) = 4.2 E452.5 − (0.264 Chlorophyll a + 0.426 Chlorophyll b)

Then, the fractions were calculated as mg/100 g fresh weight of leaves.

#### 2.5.4. Determination of Total Soluble Carbohydrates

A total of soluble carbohydrates was determined by the anthrone method [34]. Leaf ground tissue was hydrolyzed with 2.5 N HCl and centrifuged; then, 4 mL of anthrone reagent was added to each extract (200 mg anthrone in 100 mL of ice-cold 95% H_2_SO_4_). The mixture was heated in boiling water bath for 8 min, cooled rapidly on ice, and then measured for absorbance at 630 nm. Total carbohydrates were calculated using a standard curve and expressed as milligrams soluble carbohydrates/gram dry weight.

### 2.6. Metabolite Profiling Using GC/MS

From each treatment, leaves samples were collected in bulk and immediately frozen in liquid nitrogen. Samples were maintained at 80 °C until analysis. After incubating 50 g of the powdered sample in 100 mL methanol overnight and extracting the compounds with a soxhlet apparatus, the excess solvent was removed from the sample and was used for testing. Leaf extracts of *P. granatum* were analyzed by gas chromatography (Thermo Scientific, Trace GC Ultra/ISQ Single Quadrupole MS) equipped with a TG-5MS fused silica capillary column (30 m, 0.251 mm, 0.1 mm film thickness), operated at 30 °C at 10 °C/5 min, and heated to 300 °C. In GC/MS detection, an electron ionization system with ionization energy of 70 eV was used, and helium gas was used as the carrier gas at a constant flow rate of 1 mL/min. Injector and MS transfer line temperatures were set at 280 °C, and the oven temperature was programmed at 40 °C as an initial temperature (hold 3 min) and increasing by 5 °C per minute (hold 5 min). Based on the comparison of their relative retention time and mass spectra with that of the NIST, WILLY library data, all the identified components were quantitatively determined using percent relative peak area.

### 2.7. Biocompatibility Evaluation Using Plant System

#### 2.7.1. Cell Membrane Stability

Based on the method of Premchandra et al. [35], as modified by Sairam [36], the membrane stability index (MSI) was calculated using a conductivity meter. Following thorough washing with tap water and adding 10 mL of double-distilled water, leaf tissues (100 mg) were heated at 40 °C for 30 min, and electrical conductivity was measured (C1). The leaf samples were then boiled for 10 min at 100 °C in a boiling water bath, and electrical conductivity was recorded (C2). The MSI was calculated using the equation:

Membrane stability index (MSI) = [1 − (C1/ C2)] × 100

According to Dhanda et al. [37], membrane injury (MI) is calculated as the ratio of membrane injury of nHAPs-treated plants to that of control plants:

MI (%) = [1 − (MSI(treated)/MSI(control)] × 100

#### 2.7.2. DNA Fragmentation Test (Comet Assay)

The comet assay was performed as described by Juchimiuk et al. [38]. Individual soybean leaves were placed in 200 μL of cold 400 mM Tris-HCl buffer, pH 7.5. To obtain a low frequency of DNA damage in control cells, the leaf was gently sliced to release nuclei into the buffer under yellow light. Previously coated slides were covered with a mixture of 1% normal melting point (NMP) agarose and 7% low melting point (LMP) agarose at 40 °C; then, they were coverslipped and placed on ice for 5 min, after which the coverslip was removed. The slide was then covered with LMP agarose (0.5%), mounted again, and allowed to cool for five minutes. The slides were placed in a horizontal gel electrophoresis tank with freshly prepared cold electrophoresis buffer (300 mM NaOH, 1 mM EDTA, pH > 13) and incubated for 15 min. The electrophoresis was performed at 16 V, 300 mA for 30 min at 4 °C. Following this, slides were submerged in neutralization buffer (400 mM Tris-HCl, pH 7.5) and stained with ethidium bromide. (20 μg/mL) for 5 min. The samples were dipped in ice-cold distilled water, covered with a coverslip, and viewed under a fluorescence microscope with a computerized image analysis system (Komet Version 3.1, Kinetic Imaging, Liverpool, UK). For each treatment, 250 randomly selected cells were analyzed (50 cells on each of five replicate slides). Integrated intensity profiles for each cell were calculated, and comet cells were estimated to determine the range of derived parameters. To quantify the DNA damage, tail length (TL) and tail moment (TM) were evaluated. Tail length (length of DNA migration) is related directly to the DNA fragment size and presented in micrometers and calculated from the center of the cell. Based on the tail length and the DNA content of the comet tail, the tail moment was calculated.

## 3. Results

### 3.1. Characterization of Phosphorous Nanoparticles

*Synthesis of nHAPs and their UV-VIS spectroscopy profile.* A change in the color of the phosphate/PPE/CE mixture after the reaction determined the initial conformation of nHAPs formation, where the reaction mixture changed to dark brown, as shown in Figure S1 (Supplementary File), which was characterized by UV-VIS spectroscopy as shown in Figure S2 (Supplementary File), with nHAPs_PPE appearing as a broadband at 320–350 nm, while nHAPs_CE appeared as a sharp peak at 320 nm.

**Figure 1:**
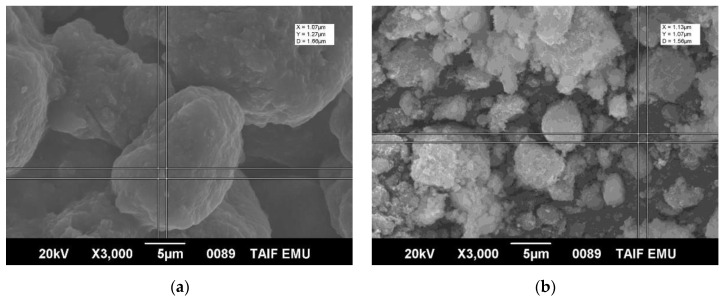
Scanning electron microscope photos showing the crystalline shape of nHAPs_PPE (**a**) and nHAPs_CE (**b**) at ×3000 magnification.

*Morphology, size, and shape of green nHAPs.* The surface morphology of the green synthesized nHAPs using PPE and CE extract was studied using SEM as shown in Figure 1a,b. SEM analysis revealed nHAP crystallites agglomerating into a large particle because of uncontrolled coagulation.

**Figure 2:**
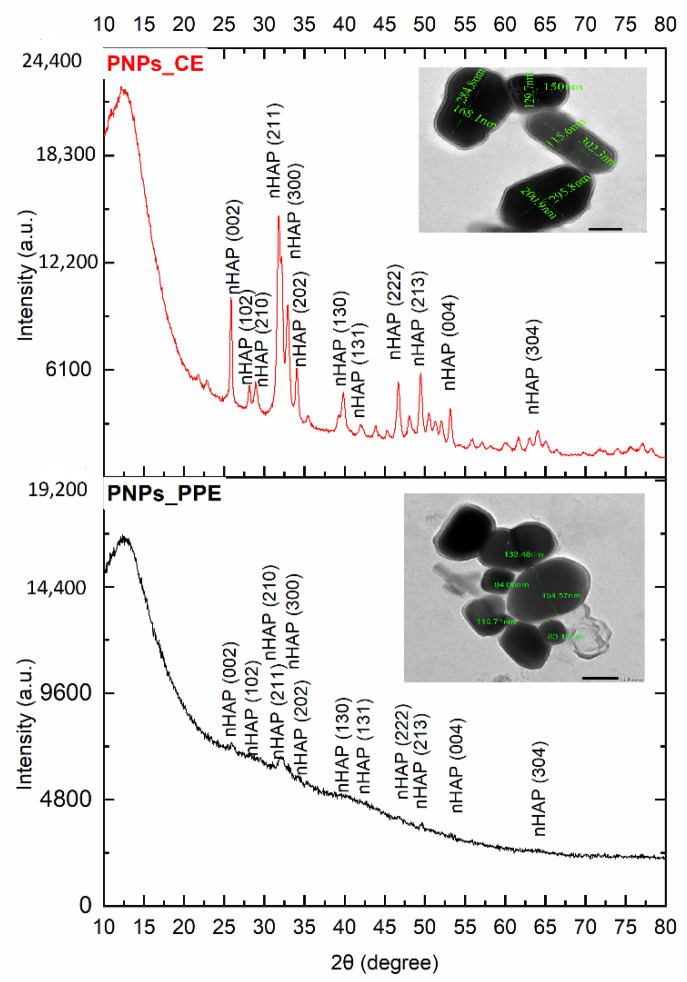
XRD patterns of nHAPs_PPE and nHAPs_CE. The top cornered TEM photomicrograph confirms the lattice shape of nanoparticles.

Figure 2 illustrates the XRD profiles of the green synthesized phosphorous nanoparticles (nHAPs_PPE and nHAPs_CE). nHAPs_CE shows more distinct and sharp peaks than nHAPs_PPE of 2θ (degree) at different planes: 25.80 (002), 28.23 (102), 28.97 (210), 31.98 (211), 32.84 (300), 33.98 (202), 39.84 (130), 42.33 (131), 46.75 (222), 49.51 (213), 53.11 (004), and 64.02 (304). According to JCPD standards with reference numbers: 89-6439, those Bragg peaks are well-coordinated hexagonal lattices of phosphorous hydroxyapatite nanoparticles. The lattice shapes of those Bragg peaks were confirmed using transmission electron microscopy (TEM). TEM indicates the average size of nHAPs_PPE (120.6 nm) and nHAPs_CE (153 nm).

**Figure 3:**
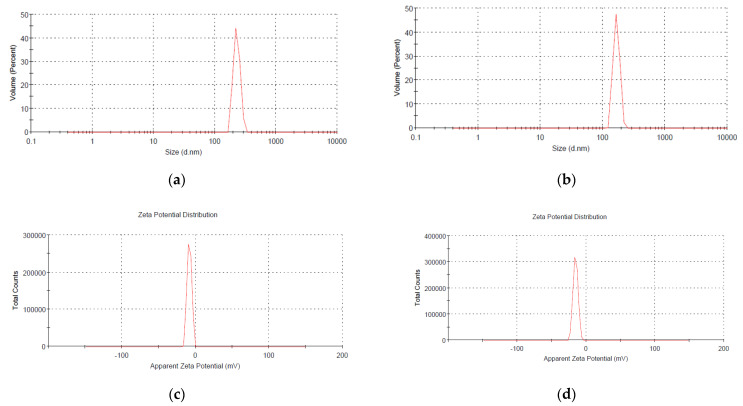
The average size of green synthesized (**a**) nHAPs_PPE (229.6 nm) and (**b**) nHAPs_CE (167.5 nm). Zeta potential of (**c**) nHAPs_PPE (−9.37 mV) and (**d**) nHAPs_CE (−16.9 mV).

Hydrodynamic size and surface charge of the synthesized PNPs were evaluated by the DLS technique. The Z-average size of nHAPs_PPE = 229.6 nm and nHAPs_CE = 167.5 nm with different zeta potentials of −9.37 mV and -16.9 mV, respectively, are shown in Figure 3.

**Figure 4:**
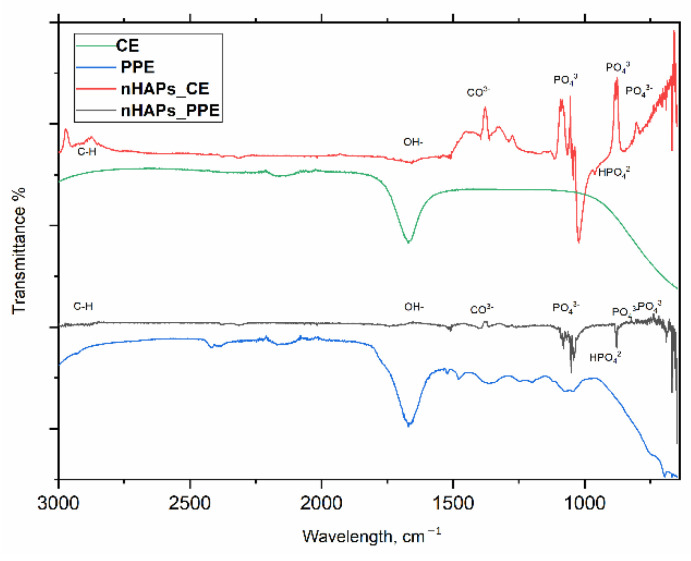
FTIR spectra of the green synthesized nHAPs_PPE and nHAPs_CE.

*FTIR analysis*. The FTIR spectroscopy was used to identify the functional groups in PPE and CE extracts that participated in the synthesis, capping, and stabilization of nHAPs. The FTIR absorption spectrum of nHAPs_PPE and nHAPs_CE is shown in Figure 4 along with their related extracts in the range of 4000 to 450 cm^−^^1^. As observed in Figure 4, the synthesized nHAPs had distinct peaks when compared to PPE and CE extracts. For both biologically formed NPs, broad peaks were assigned at 649.35 cm^−1^ and 1631.87 cm^−1^. Strong and sharp peaks were observed at 567 cm^−1^ and 620.65 cm^−1^, between 900 and 1100 cm^−1^, and at 2925 cm^−1^. In addition to the presence of peaks at 463.35 cm^−1^ and 874.66 cm^−1^, broadbands at 1402 cm^−1^ and 1460 cm^−1^ were observed.

### 3.2. Biochemical Analysis

#### 3.2.1. Photosynthetic Pigments

**Figure 5:**
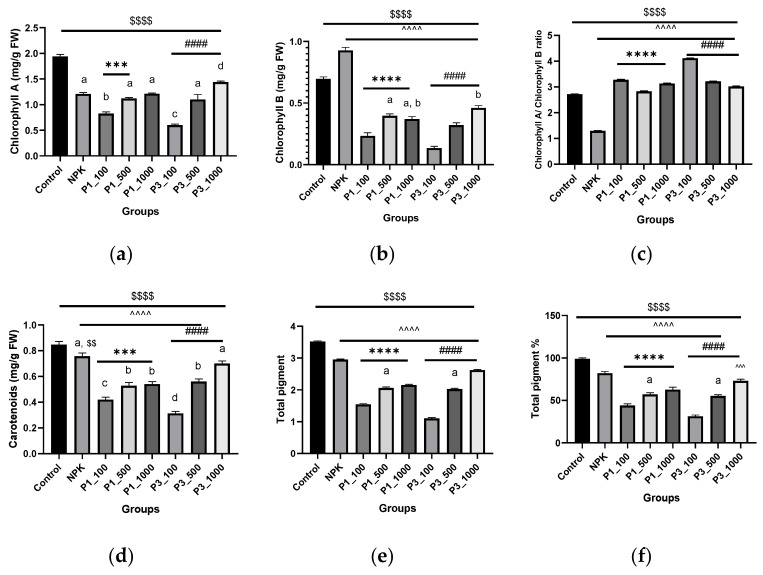
Photosynthetic pigments analysis: chlorophyll A content (**a**), chlorophyll B (**b**), chlorophyll A/chlorophyll B ratio (**c**), carotenoids content (**d**), total pigment (**e**), and percentage of the total pigment (**f**) in different groups. Results are expressed as mean ± SE, $ (dollar sign) significant difference with negative control at *p* < 0.0001, # (number sign) statistically compared P3 groups with the P3_100. ^ (circumflex accent) statistically compared with NPK. Different letters refer to statistically significant differences between groups at *p* ≤ 0.001, while the same letters refer to non-significant (ns) difference between groups at *p* ≤ 0.05, using one-way ANOVA followed by Tukey’s test. **** indicates *p* ≤ 0.0001, ***, indicates *p* ≤ 0.001 (that was applied to the other signs). P1 = nHAPs_PPE; P3 = nHAPs_CE.

Figure 5 summarizes the chlorophyll a (Chl a), chlorophyll b (Chl b), and carotenoids (Car) contents in leaves of *P. granatum* treated with green synthesized nHAPs. Based on the data collected from pomegranate plantlets grown in pot conditions, it was determined that foliar spraying nHAP at different concentrations had a substantially negative impact on photosynthetic pigments. Generally, spraying leaves with nHAP_PPE, nHAP_CE, and NPK significantly reduced total photosynthetic pigments (Chl a, b, and Car) compared to the control, with the lowest value induced by nHAP_CE at 100 mg/L. (Figure 5e,f). There was also variable significant change among pigments individually in comparison to the chemical fertilizer NPK. For example, Chl a showed a significant decrease at low doses (100 mg/L), whereas it increased approximately the same as NPK except at the higher dose of nHAP_CE (1000 mg/L). However, Chl b levels were permanently reduced in all nHAP treatments compared to control and NPK-treated plants (Figure 5b). According to Figure 5c, the ratio of Chl a/Chl b data demonstrates higher values in all nHAPs treatments with inverse dose relationships. As shown in Figure 5d, the Car pattern was similar to that of Chl a in that a decline at low concentrations was followed by an increase up to the NPK level at higher concentrations (nHAP_CE at 1000 mg/L).

#### 3.2.2. Total Soluble Carbohydrates

**Figure 6:**
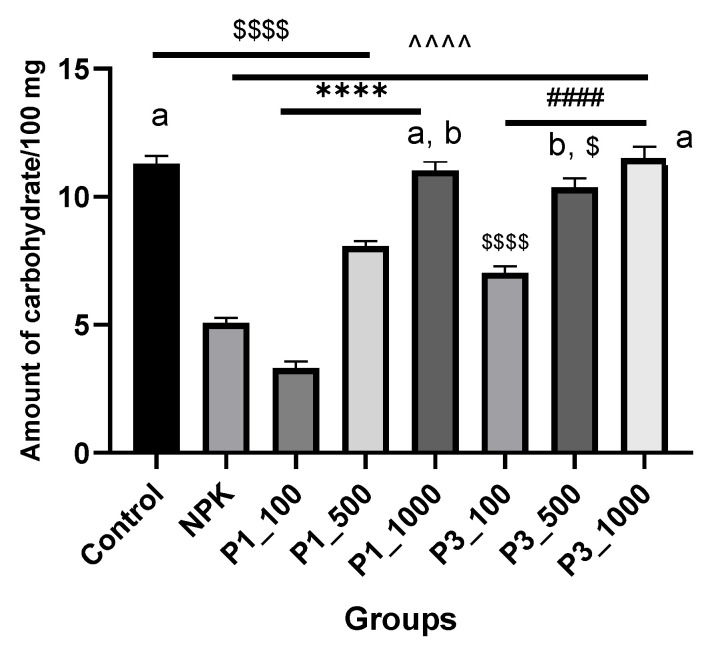
Total soluble carbohydrates in different nHAPs-treated plants. Results are expressed as mean ± SE, $ (dollar sign) significant difference with negative control at *p* < 0.0001, # (number sign) statistically compared P3 groups with the P3_100. ^ (circumflex accent) statistically compared with NPK. Different letters refer to a statistically significant difference between groups at *p* ≤ 0.001, while the same letters refer to a non-significant (ns) difference between groups at *p* ≤ 0.05, using one-way ANOVA followed by Tukey’s test. **** indicates *p* ≤ 0.0001, (that was applied to the other signs). P1 = nHAP_PPE, P3 = nHAP_CE.

With increasing concentrations of nHAPs, total carbohydrate levels were reduced similarly to photosynthetic pigments. A greater reduction in total soluble carbohydrate is indicated by the lowest dose (nHAP_100) and then increases with increasing concentrations (Figure 6). The percent of total soluble carbohydrate decreased by 70.68, 31.03%, and 39.65, 10.34% after treatments with nHAP_PPE and nHAP_CE (at 100 mg/L and 500 mg/L), respectively, as compared to control. In all the nHAPs-treated plants except for nHAP_PPE 100, total carbohydrates were significantly higher than in NPK-treated plants.

### 3.3. Metabolite Profiling

**Figure 7:**
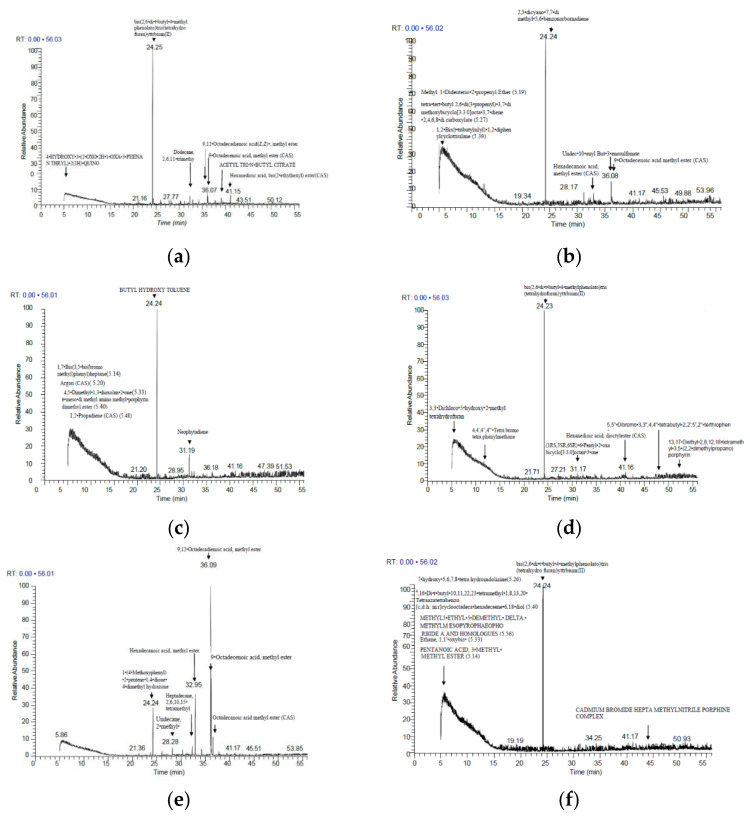
Chromatograms generated by gas chromatography-mass spectrometry (GC/MS) analysis which demonstrates metabolite profiling of *Punica granatum* leaves of the following treatments: (**a**) Control; (**b**) NPK; (**c**) nHAP_PPE50; (**d**) nHAP_PPE1000; (**e**) nHAP_CE50; (**f**) nHAP_CE1000.

From the applied concentrations of nHAPs, we chose the less (50 mg/L) and the higher (1000 mg/L) doses to examine the chemical analysis of *P. granatum* plants by GC-MS analysis. The active constituents are presented in Figure 7 and Table S1 (Supplementary File). GC-MS results revealed several compounds at various periods to determine their nature and structure in leaf extracts of *P.granatum* (Table S1, Supplementary File); peaks correlating to bioactive compounds were identified by comparing their peak retention time, peak area %, and mass spectral fragmentation patterns to those of known compounds explained in the National Institute of Standards and Technology (NIST) library. Metabolite profiling revealed the presence of mainly 20 phytochemical compounds (Table S1, Supplementary File); those identified major compounds showed noticeable variations across all treated samples compared to control. The main components of the control sample were as follows: (2,6-di-t-butyl-4-methylphenidate)tris(tetrahydrofuran)ytterbium(II) (57.44%); 4-hydroxy-3-(2.oxo-2*H*-1-phenanthryl)-2(1*H*)-quino (4.34%); 9,12-octadecane (Z,Z), methyl ester (4.94%); Hexanedioic acid, bis(2-Ethylhexyl ester (CAS) (4.10%); 9-Octadecenoic acid (3.19%), Dodecane, 2,6,11- trimethyl-(CAS) (3%), and acetyl tris-*N*- butyl citrate (2.22%).

### 3.4. Biocompatibility Assays

#### 3.4.1. Testing on Mammalian Cells Using MTT Assay

**Figure 8:**
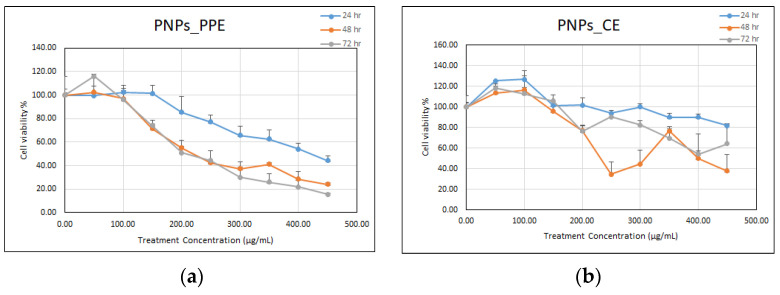
Percentage of cell viability of Vero E6 cells according to serial dilutions treatment of nHAP_PPE (**a**), and nHAP_CE (**b**) at different sampling times: 24 h, 48 h, and 72 h.

Based on the MTT assay, nHAPs_PPE and nHAPs_CE were tested for biocompatibility with mammalian cells using the Vero E6 normal cell line as a representative sample. nHAPs display cytotoxic effects against Vero cells in a time- and dose-dependent manner, as shown in Figure 8a,b. Cell viability significantly decreased after 72 h at nHAPs_PPE and nHAPs_CE concentrations of 100, 200, 300, 400, and 450 μg/ mL^−1^. To verify cytotoxicity, the NPs concentration that causes 50% growth inhibition of a cell line (IC50) was determined. Overall, the average IC50 for nHAPs_PPE and nHAPs_CE was 230 and 446 μg/mL, respectively.

#### 3.4.2. Testing on Plant Cells

**Figure 9:**
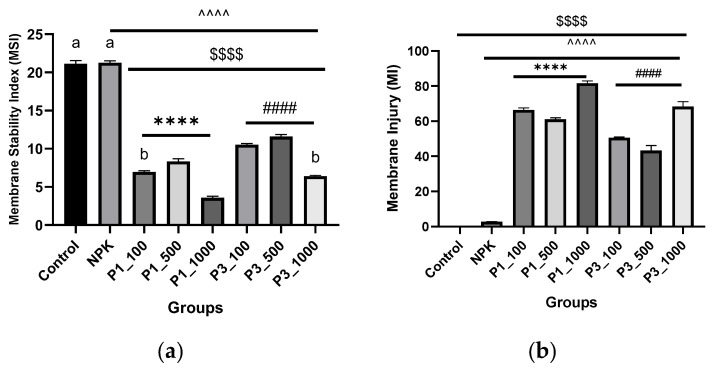
(**a**) Membrane stability index (MSI) and (**b**) membrane injury (MI) in different nHAPs-treated plants. Results are expressed as mean ± SE, $ (dollar sign) significant difference with negative control at *p* < 0.0001, # (number sign) statistically compared P3 groups with the P3_100. ^ (circumflex accent) statistically compared with NPK. Different letters refer to the statistically significant difference between groups at *p* ≤ 0.001, while the same letters refer to non-significant (ns) difference between groups at *p* ≤ 0.05, using one-way ANOVA followed by Tukey’s test. **** indicates *p* ≤ 0.0001, (that was applied to the other signs). P1 = nHAP_PPE, P3 = nHAP_CE.

**Membrane stability and cell integrity**. The results shown in Figure 9a,b demonstrate that all nHAPs treatments reduced cell integrity (MSI) and subsequently increased membrane injury (MI). The changes in membrane stability index (MSI) differed significantly between treatments (3.7 and 6.4%), while levels of membrane injury on *P. granatum* leaves treated with both nHAPs_PPE and nHAPs_CE at a higher dose (1000 mg/L) were significantly higher (82.41% and 69.91%) than in the control treatment (Figure 9a,b).

**Figure 10:**
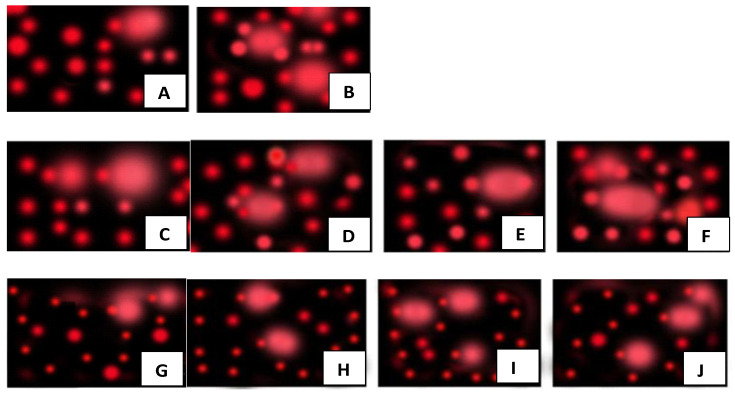
Photomicrographs of EtBr-stained DNA from protoplasts of *P. granatum* treated with nHAPs: (**A**) control; (**B**) NPK; (**C**–**F**) nHAP_PPE at concentrations of 50, 100, 500, and 1000 ppm, respectively; (**G**–**J**) nHAP_CE at concentrations of 50, 100, 500, and 1000 ppm, respectively.

**Figure 11:**
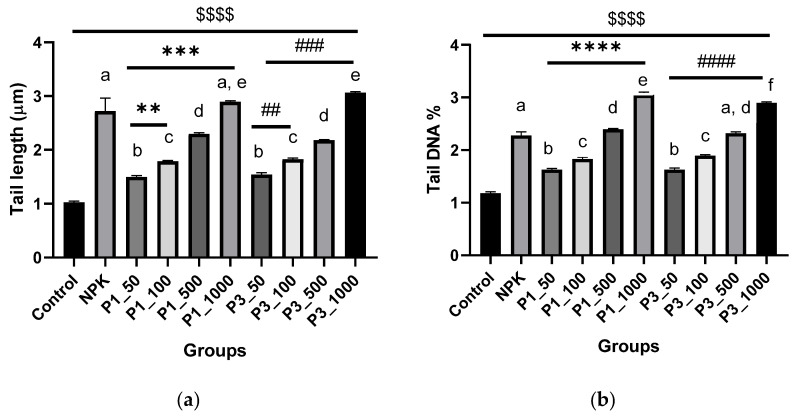

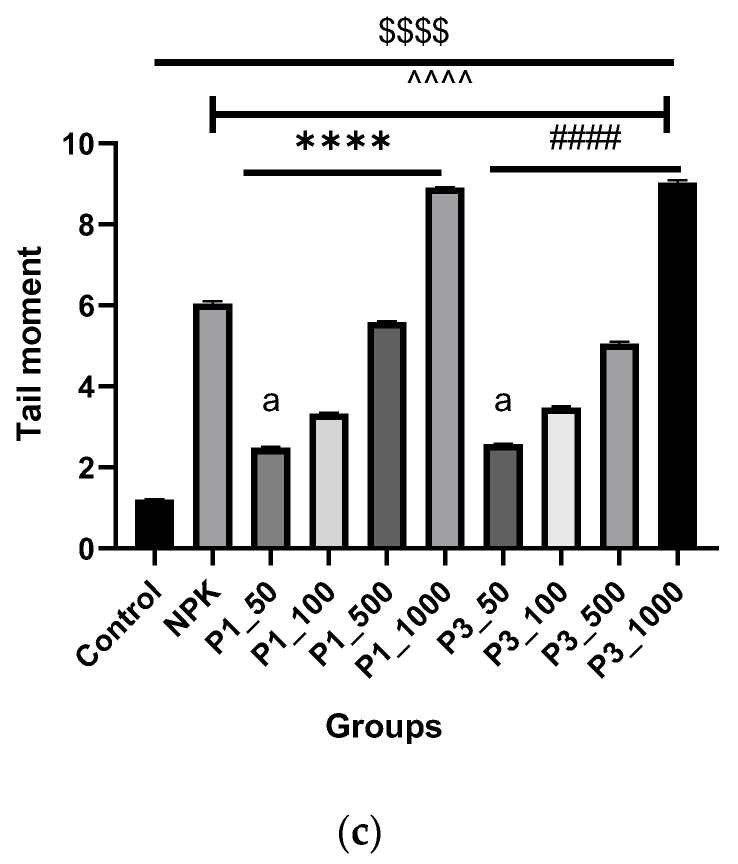
DNA damage represented as tail length (μm) (**a**), percentage of DNA in tail (**b**), and tail moment (**c**) in different groups. Results are expressed as mean ± SE, $ (dollar sign) significant difference with negative control at *p* < 0.0001, * (asterisk) statistically compared P1 (nHAP_PPE) groups with the P1_50 and # (number sign) statistically compared P3 (nHAP_CE) groups with the P3_50. ^ (circumflex accent) statistically compared with NPK. Different letters refer to statistically significant differences between groups at *p* ≤ 0.001, while the same letters refer to non-significant (ns) differences between groups at *p* ≤ 0.05, using one-way ANOVA followed by Tukey’s test. **** indicates *p* ≤ 0.0001, ***, indicates *p* ≤ 0.001, ** indicates *p* ≤ 0.01 (that was applied to the other signs). P1 = nHAP_PPE, P3 = nHAP_CE.

**Comet assay**. The results of the DNA migrations (comet assay) in pomegranate leaf samples are displayed in Figure 10. As the most useful parameters of the comet assay for evaluating DNA damage are highly variable, it was decided to measure tail length (TL), tail moment (TM), and % tail DNA (TD%). Statistical analysis indicates that the frequency of DNA damage in the nuclei of *P. granatum* increased steadily with increasing concentrations of nHAPs used, regardless of whether DNA damage was expressed as tail moment, tail DNA, or tail length (Figure 10). Based on the comet assay that determines DNA damage based on tail DNA (%), both nHAP_PPE and nHAP_CE significantly caused DNA damage in a dose-dependent manner. However, a dosage of 1000 ppm was toxic, as indicated by the comets that showed nuclear disintegration or necrosis (Figure 10). As shown in Figure 10, the two higher doses of green synthesized nHAPs (PPE and CE) were used in this study, and both significantly increased the control TD% values as follows (at *p* < 0.0001): for nHAP_PPE, it increased from 1.19 to 2.41 and 3.05, respectively, and for nHAP_CE, it increased to 2.30 and 2.92, respectively. No significant increase in DNA damage was observed in pomegranate nuclei at the lowest nHAPs dose (50 ppm). Our results, however, showed that the TD% values induced by chemical fertilizers (NPK) were similar to those of the highest nHAP concentrations (Figure 11).

## 4. Discussion

### 4.1. nHAP Preparation and Characterization

The present study reported a successful synthesis of nanohydroxyapatite using pomegranate peel and coffee grounds extracts, which was confirmed by multiple characterization techniques. A dark brown color change of PPE/CE extracts detected at the end of nanoparticle preparation is the first sign that nanoparticles have formed. The UV-VIS spectra also showed maximum absorbance at 320 nm and 320–350 nm for nHAPs_CE and nHAPs_PPE, which is in agreement with the previous study reported by Kalaiselvi et al. [39], in which Moringa oleifera flower extract was used to create hydroxyapatite nanorods by the microwave-assisted method, which was detected by UV-VIS spectroscopy at 310 nm.

nHAPs_CE demonstrated significant sharp peaks in the XRD profile, indicating a good crystallinity of the final product with hexagonal nanocrystalline structure, which was consistent with their TEM analysis. As opposed to nHAPs_CE, the XRD spectrum of nHAPs mediated by PPE showed sharper peaks, and this might be explained by differences in pH of the two extracts used. The pH of pomegranate peel extract was 4.83 [40], while that of coffee grounds extract was close to neutral (6.5 to 6.8) [41]. Changes in pH (from alkaline to acidic), temperature, and reaction time affect the degree of crystallinity [42]. This is clearly demonstrated by the present XRD investigation. Our present findings report the successful synthesis of phosphorus nanoparticles derived from hydroxyapatite, which is a compound consisting of phosphorus and calcium in a 67:1 stoichiometric ratio, as previously reported [43].

According to our findings, the higher zeta potential negativity (−16.9 mV) of nHAPs_CE is a contributing factor to their smaller sizes (167.5 nm) than nHAPs_PPE by increasing electrostatic forces between the nanoparticle surfaces that prevent them from aggregating to larger particles. This clearly appeared in the SEM micrograph that showed an agglomeration of nHAP, especially nHAPs_PPE, into a large particle due to their uncontrolled coagulation. Vasadarajan et al. [44] synthesized hydroxyapatite nanoparticles using banana leaves, tamarind leaves, prickly pear fruits, and grapes. They found that hydroxyapatite nanoparticles made from grapes have higher zeta potential negativity of -29 mV with the smallest size being 292 nm.

In the FTIR analysis, broad peaks were observed at 1631.87 cm^−1^ and 649.35 cm^−1^, respectively, due to the bending of water and OH− deformation. Those findings were consistent with those of Kalaiselvi et al. [39]. The intense broad peak between 900 and 1100 cm^−1^ was assigned to the stretching mode of PO_4_^3−^. Meanwhile, the intense sharp peaks at 620.65 cm^−1^ and 567 cm^−1^ are due to the bending modes of PO_4_^3−^ caused by hydroxyapatite formation. The weak band at about 483.35 cm^−1^ was caused by the phosphorus bending vibration, the peak at 874.66 cm^−1^ was caused by the HPO_4_^2−^ groups, and CO^3−^ derived bands appeared at 1402–1460 cm^−1^. Additionally, the peak at 2925 cm^−1^ is attributed to the C–H stretching of polyols [42]. Polyols are abundant compounds that are widely present in plant extracts (including pomegranate and coffee extracts) that are responsible for the reduction and stabilization of nanoparticles through their green synthesis. Based on these results, it could be suggested that the prepared nanoparticles are good in the assigned mode [45,46]. According to the FTIR spectrum of synthesized nHAPs, the presence of different functional groups indicates the presence of biomolecules such as flavonoids in the PPE and CE extracts during the synthesis process. These functional molecules are responsible for the capping and stabilization of nHAPs.

### 4.2. Biochemical Analysis

#### 4.2.1. Photosynthetic Pigments

Measurement of photosynthetic pigments can provide valuable information about the physiological state of plants [47,48]. Concentrations of chlorophylls and carotenoids, which participate in the absorption and transmission of light energy, are susceptible to changes caused by inorganic chemical stressors. In this study, Chl a was severely affected by all treatments, which could be explained by an increase in chlorophyll degradation. However, nHAP_CE and nHAP_PPE treatments given at the same dose did not reveal a significant variation in results. Likewise, Chl b and Car content showed a pattern of change following Chl a, indicating a change in the photosynthetic machinery via chlorophyll recycling and the presence of photosystem II employing higher concentrations [49]. The effects of almost all treatments were seen to decrease photosynthetic pigments, with the lowest Chl a, Chl b, and Car concentrations observed for the (nHAP 100) treatment. This is in agreement with Hu et al. [50], who found that the lowest levels of photosynthetic pigments were found to be obtained with nanometric ZnO at 50 mg/L. On the other hand, increased Chl a/b shows greater Chl a. It is believed that the chlorophyll a/b ratio is a measure of the antenna sizes in PS I and PS II [51]. Accordingly, the core antenna is composed of only chlorophyll a, while the outer antenna contains Chl a and Chl b. Therefore, higher Chl a/b ratios in nHAPs plantlets than control and NPK plants will indicate greater efficiency in PS I and PS II antenna complexes, leading to a better capacity of photosynthesis even under stress conditions. The results also indicated that nanoparticles may induce the pomegranate plants to tolerate stress conditions. According to Wang et al. [52] and Broadley et al. [53], the mechanism may be due to nanoparticles stimulating Mg, Fe, and Mn deficiencies, which are elements that are essential for the synthesis of chlorophyll. As well, other researchers [54,55,56,57] explained the decline of photosynthetic pigments in plants under NPs treatment, by peroxidation of the chloroplast membrane due to the aggravation of oxidative stress caused by ROS, which is mainly produced in chloroplasts and presumably mitigates stress induced by nanoparticles. Moreover, oxidative stress decreases chlorophyll because of the destruction of pigments. At the same time, chlorophyll is decomposed in the chloroplasts, and the thylakoids disappear [49].

#### 4.2.2. Total Soluble Carbohydrates

In response to oxidative stress, soluble carbohydrates accumulate, as they play an important role in maintaining and enhancing membrane stabilization by acting as reactive oxygen species (ROS) scavengers [58]. In line with the results from photosynthetic pigments analysis, NPK and nHAPs reduced the accumulation of total soluble sugars in the treated plants when compared to the control; the lowest content of total soluble sugars was observed in plants treated with 100 mg/L nHAPs. Interestingly, plants exposed to 500 and 1000 mg/L nHAP concentrations experienced significant increases, reaching the control value. The results of our study confirmed those of other findings [59,60] following treatment of potato and *Abelmoschus esculentus* plants with 100 and 500 ppm Zn_NPs. In this regard, the accumulation of soluble carbohydrates was related to enhancing plants’ resistance to nanoparticle stress, since carbohydrates have several functions in plants, such as energy storage, signaling, and adaptation to environmental stresses [61]. This explains why higher nHAP concentrations lead to higher carbohydrate fractions in pomegranate leaves.

### 4.3. Metabolite Profiling

To determine whether the two NPs could alter the metabolism of pomegranate plants, subsequent GC/MS studies were performed to identify the remarkably altered metabolites. As revealed by the chromatograms of the metabolite fingerprints, the composition of leaf metabolites in nHAP_PPE or nHAP_CE treated pomegranate plants was separated in the first principle component according to control and NPK fertilizer levels. A GC/MS analysis found that foliar spraying of nHAP_PPE or nHAP_CE affected bioactive metabolites accumulation in pomegranate plants after the application of nHAPs or NPKs. Several previous studies have also indicated that NPs alter metabolite pools in plants [62,63,64]. However, the detailed molecular mechanisms remain unclear. Those metabolites play multiple roles in plants, to modulate plant growth and development. Furthermore, results of this study indicated a dose-dependent metabolite profiling, as at nHAPs 50, the prominent effect was revealed by the change in compounds that belong to fatty acids and lipids class (e.g., 9,12•Octadecadienoic acid(Z,Z)•, methyl ester and octadecane (CAS), which are considered amongst the most important fatty acids in pomegranate plants [65]. Likewise, polyphenolic compounds and derivatives (e.g., bis2,6-dinitrophenyl-4-methylphenol and bis(tetrahydrofuran)ytterbium(II)) that play a role in plant defense/stress responses appeared downregulated in plants sprayed with a high concentration (1000 mg/L) of nHAP_PPE (45.15%) and nHAP_CE (34.65%), as compared to control plants (57.44%). These findings are consistent with those of Li et al. [66], who demonstrated that foliar exposure to NPs altered metabolic processes in cucumber plants, especially leaves. Elevated levels of secondary metabolites suggest that nHAPs may regulate biotic stress resistance in plants, which is a hypothesis that needs further investigation. Consequently, future studies will examine the entire plant life cycle and focus on fruit development in order to find out how both NPs affect yield and quality.

### 4.4. Biocompatibility with the Biological Systems

#### 4.4.1. MTT Assay with Mammalian Cells

Hydroxyapatite nanoparticles were used for various medical and cosmetic purposes. In addition, hydroxyapatite nanoparticles were primarily used as a nano-fertilizer for plants to contain phosphorus, which is one of three main components of the NPK (nitrogen–phosphorus–potassium) fertilizers [67]. Therefore, cytotoxicity studies are necessary to assess their effects on mammalian cells. Results have shown that nHAPs_CE is a safer form of nano-hydroxyapatite with an IC50 of 446 μg/mL on normal Vero E6 cells than that prepared from PPE extract. These conclusions are in line with an earlier study by Ramis et al. [68], which found that 3.1% nano-hydroxyapatite had no adverse effects on the human gingival epithelium (HGE) tissue model using MTT assay. Moreover, previous results indicated that hydroxyapatite nanoparticles did not induce cytotoxicity up to 800 mg/mL in mouse bone marrow mesenchymal stem cells using MTT assay [69] and <300 mg cm^−2^ in primary rat hepatocyte cultures, using MTT and LDH assays [70].

#### 4.4.2. Biocompatibility Assays of nHAPs with *Punica granatum* Plants

Membranes are the first place in a cell to be influenced by stress conditions, so the ability of plants to maintain membrane integrity under abiotic stress determines their tolerance to that stress. A membrane stability index measures the extent of the damage and describes the ability of a membrane to withstand stress. Using the membrane stability index, one can measure the percentage of damage to a membrane and evaluate the ability of the membrane to withstand stress conditions. As a result of lipid peroxidation, membranes are damaged and selective permeability of the membrane is disrupted, which results in cellular permeability and electrolyte leakage [71]. A foliar application of nHAPs lowered the membrane stability and increased the membrane injury of *Punica* cells in treated plants. The production of reactive oxygen species destroys the structure of the cellular membrane causing electrolyte leakage. nHAP_PPE also caused significantly more damage to the membrane when compared to nHAP_CE. These results agree with results obtained with MTT.

Nano-hydroxyapatite nanoparticles (nHAPs) have been suggested to provide phosphorus nutrition to plants as a nano-fertilizer; however, some reports indicate that those NPs might negatively affect plant growth [72]. While these studies are in their infancy yet, there has never been a proposal for their macro-consumption. Several studies have questioned the cost-effectiveness and efficiency of nano-fertilizers, so it was important to investigate the effects of nHAPs on target crop plants as well as mammalian cells. As research has become increasingly concerned about the adverse effects of nanoparticles on biological systems [73], many scientists have measured the genotoxic effects of NPs [74,75,76,77]. In the context of following the OECD guidelines on nanotoxicity assessment, DNA damage can be detected and characterized not only through direct interactions such as the detection of DNA breaks and altered DNA bases but also indirectly and indirectly via secondary mechanisms (e.g., oxidative stress). As it is simple and sensitive, single-cell gel electrophoresis (SCGE), commonly known as ‘Comet assay,’ meets these criteria and may therefore be suitable to include in upcoming guidelines on nanotoxicity assessment. It measures strand breaks in DNA at the level of individual eukaryotic cells [78]. It is commonly performed in animal cells, either in culture or isolated from the organism, while it is possible to examine DNA damage in plant cells as well [79]. In addition to its high sensitivity, the alkaline comet method shows several advantages including its low cost, flexibility, performance capability, and short assay execution time. Furthermore, it allows the measurement of DNA damage during any phase of the cell cycle, since it considers both DNA content and DNA damage [80]. An image analysis technique for individual cells is a very suitable approach for comet scoring and analysis [80,81]. There are other parameters [82] that are just as useful such as tail length, the relative fluorescence intensity of tail (normally expressed as a percentage of DNA in the tail), and tail moment (the product of DNA in the tail and tail intensity, which are not based on image analysis). A direct correlation has been observed between the degree of intensity of a comet tail and the amount of DNA strand breaks in individual cells [78].

The genotoxicity of different NPs in plants is widely studied using comet assays, and the results are usually expressed in terms of percentage of tail DNA [83,84]. To the best of our knowledge, limited studies have been conducted to evaluate the genotoxicity of nHAPs in plant cells. In this study, treatments with nHAPs (100, 500, 1000 mg/L) caused significant increases in DNA damage in leaves, especially after higher concentration treatments. At the same time, NPK conventional fertilizer still caused a higher level of DNA damage than nHAPs. The plants sprayed with nHAP_CE were somewhat less sensitive to changes in DNA than those sprayed with nHAPs_PPE. These findings are consistent with the results of other biocompatibility assays in this study, which proved that nHAPs_CE could be safer for applications. Our comet assay results showed that a low concentration at 50 mg/L is not genotoxic in *Punica* plants. This is in line with earlier reports stating that Fe3O4 NP low concentrations up to 70 mg/L are not toxic to plant cells [85,86].

All the data in this study support the negative impact of nHAPs on *P. granatum* plants. Accordingly, the chemical structure of nHAPs and increased Ca^2+^ concentration in plant cells may explain this phenomenon [72]. Consequently, biological interactions of nHAPs with plants may vary as well as their toxicity. Therefore, further studies are needed to understand how nHAPs are absorbed by various plants and cells. In the literature, there have been contradictory results reported concerning the use of nHAPs as nano-fertilizers. Positive effects have been observed on many crops (e.g., soybeans, sorghums, peas, and pak choi) [72]. On the other hand, other plants (e.g., mung bean sprouts, tomato, and pak choi) responded negatively to nHAPs. The findings of Li et al. [87] suggest that a P-nano-fertilizer (nHA) might be more effective as a soil amendment than as a foliar application. A carrier material, such as a biopolymer, could, however, significantly increase the efficacy of the nHA.

## 5. Conclusions

Hydroxyapatite nanoparticles (nHAPs) have been tested for their potential applications in the phosphorus nutrition of plants, and recent reports have shown inconsistent results about their effects on plants. Some researchers found that the nanoparticles were beneficial, while others reported negative effects. Reports showing the negative effects of these nanoparticles on plants cannot be ignored. Divergent results could be caused by species of plants, NP properties (size, shape, type, structure and defect, surface coating, etc.), and culture environment, and there are still a lot of controversies and problems that need to be resolved. Although our green ecofriendly approach was successful in creating hydroxyapatite phosphorous nanoparticles, the results of this study did not support their use as nano-fertilizers. Relying on the current detection methods is not enough, and it is imperative that nHAPs be used correctly to eliminate negative effects and achieve positive effects. As a result of the dose- and time-dependent limitations observed in experimental conditions, the use of nHAPs in crop management should be more cautious. On the other hand, it is vital to fully comprehend the phytotoxic mechanisms of NPs before it is applied in field environments; a method of reducing that toxicity must also be developed. Therefore, to improve the research on the bioavailability of nanoparticles by plants, it is necessary to conduct long-term dose exposure studies. In addition, the diversity of material exposure is also essential, such as incorporating short-term exposures at high concentrations into long-term exposures at low concentrations.

## Data Availability

All data generated or analyzed during this study are included in this published article.

## Supplementary Materials

The following supporting information can be downloaded at: https://www.mdpi.com/article/10.3390/nano12091527/s1, Figure S1. Preparation of phosphorous nanoparticles biologically using pomegranate peel extract (nHAPs_PPE) and coffee ground extract (nHAPs_CE), Figure S2. UV-Visible spectral analysis of green synthesized nHAPs_PPE and nHAPs_CE, Table S1. GC/MS results of the ethanolic extract of *Punica granatum* leaves under study: RT (retention time); MW (molecular weight); MF (molecular formula).
