# Supplementary material for: Green Synthesis of Phosphorous-Containing Hydroxyapatite Nanoparticles (nHAP) as a Novel Nano-Fertilizer: Preliminary Assessment on Pomegranate (Punica granatum L.)
Source: Nanomaterials (Basel). 2022 May 1;12(9):1527. doi: 10.3390/nano12091527 (PMC9101472; doi:10.3390/nano12091527)
Supplement: Supplementary file 1 [file nanomaterials-12-01527-s001.zip › nanomaterials-1673934-supplementary.pdf]

Article

Green Synthesis of Phosphorous-Containing Hydroxyapatite Nanoparticles (nHAP) as a Novel Nano-Fertilizer: Preliminary Assessment on Pomegranate (*Punica granatum* L.)

Hala M. Abdelmigid [1,*], Maissa M. Morsi [2], Nahed Ahmed Hussien [2,*], Amal Ahmed Alyamani [1], Nawal Abdallah Alhuthal [3] and Salim Albukhaty [4,*]

1 Department of Biotechnology, College of Science, Taif University, P.O. Box 11099, Taif 21944, Saudi Arabia; a.yamani@tu.edu.sa
2 Department of Biology, College of Science, Taif University, P.O. Box 11099, Taif 21944, Saudi Arabia;
m.moasa@tu.edu.sa
3 Department of Chemistry, College of Science, Taif University, P.O. Box 11099, Taif 21944, Saudi Arabia; nawal.h@tu.edu.sa
4 Department of Chemistry, College of Science, University of Misan, Maysan 62001, Iraq
* Correspondence: h.majed@tu.edu.sa (H.M.A.); n.nahed@tu.edu.sa (N.A.H.); albukhaty.salim@uomisan.edu.iq (S.A.)

Table S1. GC/ MS results of the ethanolic extract of *Punica granatum* leaves under study: RT (retention time); MW (molecular weight); MF (molecular formula).

Treatment	RT	MW	MF	AREA%	Probabilities of the Detected Compounds
Control	5.18	355	$C_{22}H_{13}NO_4$	5.43	4-Hydroxy-3-(2-oxo-2*H*-1-oxa-3-phenanthryl)-2(1*H*)-quinolinone
	11.90	436	$C_{31}H_{64}$	1.03	Hentriacontane (CAS)
	18.24	254	$C_{18}H_{38}$	1.11	Octadecane (CAS)
	21.17	126	$C_8H_{14}O$	1.21	1-Octen-3-one (CAS)
	24.24	828	$C_{42}H_{70}O_5Yb$	57.44	bis(2,6-di-t-butyl-4 methylphenoxo)ytterbium(II)
	24.39	206	$C_{16}H_{14}$	2.16	trans, trans-1,4-Diphenyl-1,3-butadiene
	25.94	224	$C1_6H_{32}$	1.54	7-Hexadecene, (Z)-
	27.77	218	$C_{15}H_{22}O$	1.95	aR-Turmerone
	28.28	254	$C_{18}H_{38}$	1.75	Hexadecane, 7,9-dimethyl (CAS)
	30.23	414	$C_{20}H_{41}Cl_3Si$	1.24	Silane, trichloroeicosyl-
	30.36	212	$C_{15}H_{32}$	1.20	Pentadecane (CAS)
	32.34	212	$C_{15}H_{32}$	3.00	Dodecane, 2,6,11-trimethyl-
	32.95	298	$C_{19}H_{38}O_2$	1.67	Octadecanoic acid, methyl ester (CAS)
	34.23	172	$C_{11}H_{24}O$	2.05	Ether, hexyl pentyl (CAS)
	36.07	294	$C_{19}H_{34}O_2$	4.94	9,12-Octadecadienoic acid (Z, Z)-, methyl ester
	36.17	296	$C_{19}H_{36}O_2$	3.19	9-Octadecenoic acid, methyl ester, (E)-
	37.78	635	$C_{18}H_{10}Br_5N$	1.48	Tris(2,4-dibromophenyl) amine
	39.11	404	$C_{20}H_{36}O_8$	2.22	Tributyl 2-acetylcitrate
	39.45	310	$C_{22}H_{46}$	1.31	Docosane (CAS)
	41.16	370	$C_{22}H_{42}O_4$	4.10	Hexanedioic acid, bis(2-ethylhexyl) ester (CAS)

Table S1. Continued.

Treatment	RT	MW	MF	AREA%	Probabilities of the Detected Compounds
NPK	5.19	72	$C_4H_6D_2O$	12.97	Methyl 1-Dideuterio-2-propenyl ether
	5.27	646	$C_{36}H_{54}O_{10}$	9.32	Tetra-tert-butyl 2,6-di(3-propenyl)-3,7-dim ethoxybicyclo [3.3.0] octa-3,7-diene-2,4,6,8-di

					carboxylate
	5.39	638	$C_{36}H_{66}Si_5$	6.52	1,2-Bis(t-tributylsilyl)-1,2-diphenylcyclotrisilane
	5.70	640	$C_{36}H_{37}BrO_4Si$	1.82	15-Bromo-4,4'-bis(t-butyl)-11,12-diethylnaphtho[12-f] phenanthrol[2,1-d]- (1, 3,2)-dioxasilepine-10,13-dione
	6.07	648	$C_{44}H_{32}N_4O_2$	1.36	meso-Tetraphenyl-2,3-cis- dihydroxy-2,3-chlorin
	10.14	578	$C_{20}H_{22}Br4$	1.26	2,2',5,5'-Tetrabromo-4,4'- di-tert-butyl bi phenyl
	24.23	220	$C_{15}H_{12}N_2$	36.33	2,3-dicyano-7,7-di methyl-5,6-benzonorbornadiene
	31.18	278	$C_{20}H_{38}$	2.64	3-Eicosyne
	32.36	671	$C_{32}H_{57}NO_6Sn$	2.34	tert-Butyl 3-Tributylstannyl-4- (methoxy ethoxy) -N-(tert-butoxy carbonyl) tyrosine
	32.96	270	$C_{17}H_{34}O_2$	2.70	Hexadecanoic acid, methyl ester (CAS)
	36.08	288	$C_{15}H_{28}O_3S$	5.45	Undec-10-enyl But-3-enesulfonate
	36.18	296	$C_{19}H_{36}O_2$	3.97	9-Octadecenoic acid methyl ester (CAS)
	41.17	370	$C_{22}H_{42}O_4$	1.66	Hexanedioic acid, bis(2-Ethylhexyl) ester (CAS)
	42.59	394	$C_{28}H_{58}$	1.51	Octacosane (CAS)
	43.51	592	$C_{36}H_{32}O_8$	1.57	1,5-bis[(6-methoxyphenyl) methyl-1,3-benzodioxol-
	45.54	228	$C_5H_8O_6S_2$	2.51	3,9-Dioxo-2,4,8,10-tetrao xa-3,9-dithiaspiro [5.5] undecane
	46.60	615	$C_{27}H_{25}C_{14}NO_7$	1.55	{4-[2'-[2''-(4'''-<Methoxycarbonyl>-2''',3'''-dichloro phenyl)-3''-ethyl-5''-pyrrolyl] methyl] butyryl]-2,3-di chlorophenoxy]-acetic acid
	46.77	116	$C_7H_{16}O$	1.37	3-Pentanol, 2,4-dimethyl-
	46.90	210	$C_{15}H_{30}$	1.77	2,4,6,8-Tetramethyl-1-undecene
	54.13	383	$C_{19}H_{30}NO_5P$	1.37	Diethyl 5-[(isopropyloxy)carbonyl]-5-methyl-2-phenylte tra hydro-1*H*-pyrrol-3yl}phosphonate

Treatment	RT	MW	MF	AREA%	Probabilities of the Detected Compounds
nHAP_PPE 50	5.14	620	$C_{23}H_{28}Br_4$	7.22	1,7-Bis (3,5-bis (bromomethyl)phenyl) heptane
	5.20	40	Ar	6.76	Argon (CAS)
	5.33	116	$C_5H_8O_3$	8.25	4,5-Dimethyl-1,3-dioxolan-2-one
	5.40	651	$C_{39}H_{49}N_5O_4$	4.08	ë-meso-di methylamino methyl-porphyrin
	5.48	40	C_3H_4	4.97	1,2-Propadiene (CAS)
	5.56	660	$C_{36}H_{44}FN_4NbO$	1.95	Fluoro(2,3,7,8,12,13,17,1 8-octaethylporphyrinato)oxoniobium
	5.82	543	$C_{31}H_{30}ClN_3O_4$	1.66	C-CAM-3-cyanomethyl Ether
	7.25	660	$C_{29}H_{26}Br_2O_8$	1.53	3',5'-Dimethoxyphenyl 1,8-Dibomo-4,5-diisoprop oxyanthraquinone-2-carboxylate
	8.00	84	CH_2Cl_2	2.09	Methane, dichloro- (CAS)
	10.98	630	$C_{42}H_{31}ClN_2O_2$	2.05	1-methyl-2,2-diphenyl-3-oxo-6-chloro-5-(5')- (1'-methyl-2',2'-diphenyl-3'-oxo1'-benzazolyl) -1-benzazole
	24.24	220	$C_{15}H_{24}O$	37.29	Butylated Hydroxytoluene
	31.18	278	$C_{20}H_{38}$	6.18	Neophytadiene
	32.35	212	$C_{15}H_{32}$	1.55	Pentadecane (CAS)
	36.08	294	$C_{19}H_{34}O_2$	1.61	9,12-Octadecadienoic acid, methyl ester, (E,E)- (CAS)
	36.18	296	$C_{19}H_{36}O_2$	1.83	9-Octadecenoic acid (Z)-, methyl ester (CAS)
	41.16	196	$C_{13}H_{24}O$	2.08	Cyclobutanone, 2-(2,6-dimethylheptyl)-
	42.59	603	$C_{38}H_{32}ClF_2N_3$	2.75	4-(4-Chlorophenyl)-2-(2- phenylethyl)-6-[4- [bis

					(4-f luorophenyl) methyl] piper azinyl-1-yl]benzonitrile
	51.83	731	$C_{39}H_{57}NO_3Ti_3$	1.86	[Tri {Titanium-penta methyl cyclo penta dienyl(ae-oxa)}(ae-methyl){*N*-(2,6-dimethyl phenyl)}]
	51.92	597	$C_{32}H_{39}NO_{10}$	2.72	3-Pyridinecarboxylic acid2,7,10-tris(acetyloxy)-1,1 a,2,3,4,6,7,10,11,11a-deca hydro-1,1,3,6,9-penta methyl-4-oxo-4a,7a-epoxy-5 *H*-cyclopenta[a]cyclopropa[f]cycloundecen-11-yl ester
	52.61	584	$C_{34}H_{68}O_5Si$	1.55	Glycerine-1,3-dimyristate , 2-O-trimethylsilyl-

Table S1. Continued.

Treatment	RT	MW	MF	AREA %	Probabilities of the Detected Compounds
nHAP_PPE1000	5.26	170	$C_5H_8Cl_2O_2$	28.66	3,3-Dichloro-5-hydroxy-2-methyltetrahydrofuran
	5.86	646	$C_{39}H_{48}N_4NiO$	1.46	Nickel(II) ç-meso-(2-Formylvinyl)octaethylchlorin
	11.36	632	$C_{25}H_{16}Br_4$	1.76	4,4',4'',4'''-Tetra bromo tetra phenylmethane
	21.72	601	$C_{31}H_{21}ClFN_3O_3S_2$	1.14	7-{4'-[4''-(5'''-Chloro-2'''-methoxybenzoyl) amino]phenyl} -2-(thienylmethylenenyl}-2-(thienylmethylenel)-2,3-dihydro-5*H*-thiazolo[3,2-a] pyrimidine
	24.23	828	$C_{42}H_{70}O_5Yb$	45.15	bis(2,6-di-t-butyl-4-methylphenolato)tris(tetrahydrofuran)yttrbium(II)
	31.18	196	$C_{12}H_{20}O_2$	1.67	(1RS,5SR,6SR)-6-Pentyl-2-oxabicyclo[3.3.0]octan-3-one
	32.35	184	$C_{13}H_{28}$	1.18	Decane, 2,6,8-trimethyl- (CAS)
	36.08	138	$C_{10}H_{18}$	1.52	cis-Pinane
	40.40	478	$C_{52}H_{60}O_4$	1.21	26,28-Dihydroxy-25,27-dioxaocta-4-ene-2,6-diynyl-p-tert-butylcalix[4]arene
	41.16	370	$C_{22}H_{42}O_4$	2.18	Hexanedioic acid, dioctylester (CAS)
	42.60	635	$C_{18}H_{10}Br_5N$	1.33	(4-Bromophenyl)bis(2,4-dibromophenyl)amine
	48.02	628	$C_{28}H_{38}Br_2S_3$	2.46	5,5''-Dibromo-3,3'',4,4''-tetrabutyl-2,2':5',2''-terthiophene
	48.62	615	$C_{27}H_{25}Cl_4NO_7$	1.22	{4-[2'-[2''-(4'''-<Methoxycarbonyl>-2''',3'''-dichlorophenyl)-3''-ethyl-5''-pyrrolyl]methyl]butyryl]-2,3-dichlorophenoxy]-aceticacid
	49.90	648	$C_{35}H_{38}Cl_2N_4O_4$	1.36	2,4-bis(á-chloroethyl)-6,7-bis[á-methoxycarbonylethyl]-1,3,5-trimethylporphyrin
	50.24	599	$C_{36}H_{44}N_4OV$	1.19	Vanadyl octaethylporphyrin
	50.36	658	$C_{42}H_{58}O_6$	1.42	Fucoxanthin
	51.06	596	$C_{40}H_{52}O_4$	1.17	Astaxanthin
	52.14	490	$C_{33}H_{38}N_4$	1.51	13,17-Diethyl-2,8,12,18-tetramethyl-3,5-(2,2-dimethylpropano)porphyrin
	52.95	713	$C_{38}H_{43}N_5O_5Zn$	1.29	{[3Z)-2-[(Dimethylcarbamoyl)methyl]-3-ethylidene-13,17-bis[2'-(methoxycarbonyl)ethyl]-2,7,12,18-tetramethyl-2,3-dihydroporphytinato]} zinc (II)
	53.87	628	$C_{28}H_{38}Br_2S_3$	1.13	5,5''-Di bromo-3,3'',4,4''-tetra butyl-2,2':5',2''-terthiophene

Table S1. Continued.

Treatment	RT	MW	MF	AREA%	Probabilities of the Detected Compounds
nHAP_CE50	5.11	177	$C_7H_{15}NS_2$	0.98	5,6-dihydro-2-ethyl-4,6-dimethyl-4*H*-1,3,5-dithiazi imethyl-4*H*-1,3,5-dithiazine
	5.18	130	$C_8H_{18}O$	1.62	2-Hexanol, 2,3-dimethyl-(CAS)
	21.36	366	$C_{26}H_{54}$	0.53	Hexacosane (CAS)
	23.79	156	$C_{11}H_{24}$	0.73	Undecane (CAS)
	24.24	246	$C_{14}H_{18}N_2O_2$	9.01	1-(4-Methoxyphenyl)-2-pentene-1,4-dione-4-dimet hylhydrazone
	26.09	212	$C_{15}H_{32}$	0.85	Pentadecane
	28.28	170	$C_{12}H_{26}$	1.63	Undecane, 2-methyl- (CAS)
	30.36	226	$C_{16}H_{34}$	1.22	2,6,10 - trimethyl - tridecane (WITHOUT stereochemistry)
	31.19	610	$C_{26}H_{16}Cl_4O_5P_2$	0.66	1,8-di (dichloro-phosphiny lidenoxy)-9,9-diphenyl-1-anthrone
	32.35	296	$C_{21}H_{44}$	1.79	Heptadecane, 2,6,10,15-tetramethyl-(CAS)
	32.95	270	$C_{17}H_{34}O_2$	12.07	Hexadecanoic acid, methyl ester (CAS)
	34.24	296	$C_{21}H_{44}$	1.57	Heptadecane, 2,6,10,15-tetramethyl-(CAS)
	36.09	294	$C_{19}H_{34}O_2$	35.77	9,12-Octadecadienoic acid, methyl ester
	36.18	296	$C_{19}H_{36}O_2$	24.25	9-Octadecenoic acid, methyl ester
	36.63	298	$C_{19}H_{38}O_2$	3.97	Octadecanoic acid methyl ester (CAS)
	37.79	310	$C_{22}H_{46}$	0.88	Docosane (CAS)
	39.46	618	$C_{44}H_{90}$	0.69	Tetratetracontane (CAS)
	45.51	128	C_9H_{20}	0.54	Hexane, 2,4,4-trimethyl-
	48.23	579	$C_{37}H_{30}N_3O_2P$	0.63	Ethyl5,6-Diphenyl -3-(triphenyl lphosphoranylideneamino) pyridazine-4-carboxylate
	54.18	83	C_4H_5NO	0.61	Isoxazole, 5-methyl-(CAS)

Table S1. Continued.

Treatment	RT	MW	MF	AREA%	Probabilities of the Detected Compounds
nHAP_CE 1000	5.14	130	$C_7H_{14}O_2$	6.11	Pentanoic acid, 3-methyl-, methyl ester
	5.26	137	$C_8H_{11}NO$	11.53	7-hydroxy-5,6,7,8-tetra hydroindolizine
	5.33	74	$C_4H_{10}O$	6.89	Ethane, 1,1'-oxybis-
	5.40	612	$C_{40}H_{44}N_4O_2$	11.30	4,16-Di-t-butyl-10,11,22,23-tetramethyl-1,8,13,20-te traazatetrabenzo[c,d:h: m:r]cyclooctadeca-hexadecaene-6,18-diol
	5.56	606	$C_{38}H_{46}N_4O_3$	9.14	Methyl 5-ethyl-5- dimethyl-delta-methyl Mesopyrophaeopho rbide A and homologues
	5.84	644	$C_{41}H_{40}O_7$	1.30	5''-(1,1-Dimethylethyl) -2 2',2'',2''',2''''-penta methoxy [1,1':3',1'':3'',1''':3'''.1''-quinquephenyl] -3,3''''-dicarboxyaldehye
	17.64	628	$C_{28}H_{38}Br_2S_3$	1.49	5,5''-Dibromo-3,3'',4,4''-tetrabutyl-2,2':5',2''-terthiophene
	21.59	618	$C_{16}H_{50}BF_3N_6Si_8$	1.28	Fluorobis [3-fluorodimethylsilyl-2,2,4,4,6,6-hexa methyl-1,3,5-triaza-2,4,6-trisilacyclohexyl] borane
	23.16	1036	$C_{67}H_{48}N_4O_4Zn$	1.45	(R, S)-{5-[4(e)-(2-(1,4,5,8,9,10-Hexahydro-1,4,5,8-tetraoxo-9,10-(o-benzeno) anthracenyl) cyclohex-(e)-y l] -10,15,20-tri-p-tolyl porphyrinato} zinc (II)
	24.23	828	$C_{42}H_{70}O_5Yb$	34.65	bis(2,6-di-t-butyl-4-methylphenolato) tris(tetrahydrofuran)yttrbium (II)
	24.38	609	$C_{37}H_{47}N_5O_3$	1.42	5-methoxy-15-nitro-2,3,7,8,12,13,17,18-octaethylporphyrin
	24.83	748	$C_{52}H_{60}O_4$	1.45	26,28-Dihydroxy-25,27-d ioxaocta-4-ene-2,6-diynyl-p-tert-butylcalix [4] arene
	26.28	726	$C_{21}H_{27}Mo_2O_6P_5$	1.29	Mo $(CO)_2[(C_4H_9\text{-}C\text{-}P)\ P_2]$ Mo $(CO)_4[(C_4H9\text{-}C\text{-}P)_2]$

31.18	156	$C_{10}H_{20}O$	1.26	(2,4,6-Trimethylcyclohexyl) methanol
32.34	170	$C_{11}H_{22}O$	1.28	Octyl Allyl Ether
36.63	632	$C_{25}H_{16}Br_4$	1.28	4,4',4",4'"-Tetrabromotetraphenylmethane
41.16	370	$C_{22}H_{42}O_4$	1.91	Hexanedioic acid, dioctyl ester (CAS)
44.08	621	$C_{27}H_{34}BrCdN_5$	1.93	Cadmium bromide hepta methylnitrite porphine complex
52.04	640	$C_{16}H_4Br_4S_4$	1.39	2,7,12,17-tetrabrom-(all- às) cyclotetrathiophen (2,7,12,17-tetrabrom Cyclo octa[1,2-b:4,3-b':5,6-b":8,7-b'"]tetrathiophen

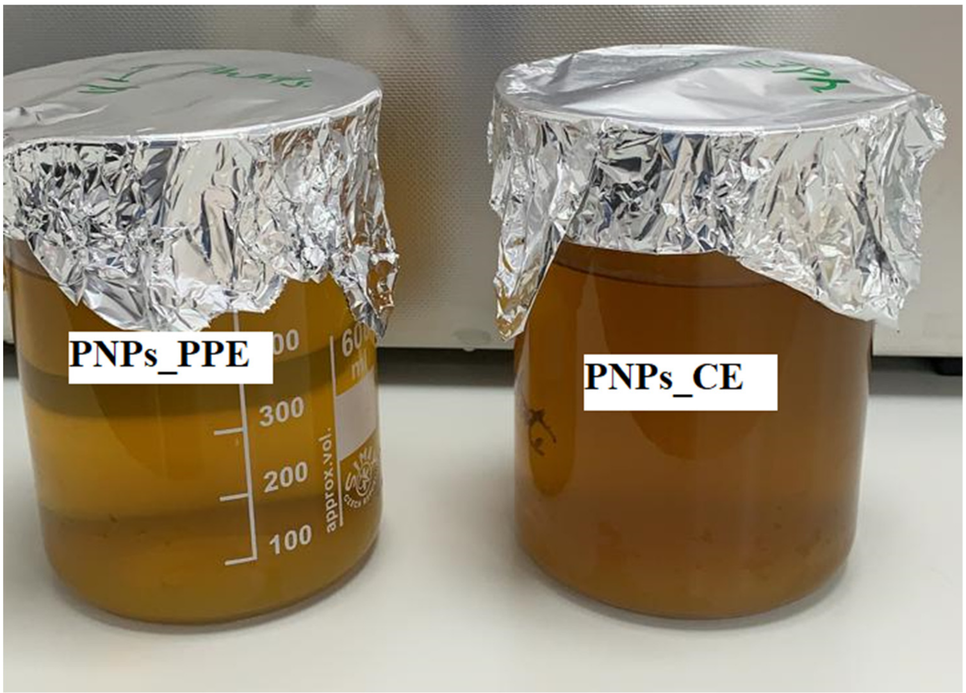

Figure S1. Preparation of phosphorous nanoparticles biologically using pomegranate peel extract (nHAPs_PPE) and coffee ground extract (nHAPs_CE).

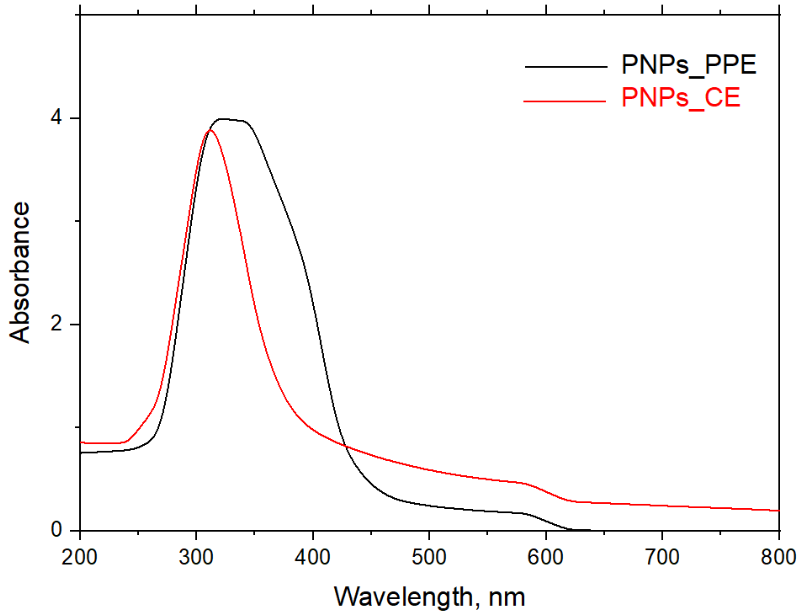

Figure S2. UV-Visible spectral analysis of green synthesized nHAPs_PPE and nHAPs_CE.
